# Illinois State Board of Public Charities

**Published:** 1883-12

**Authors:** Fred. H. Wines

**Affiliations:** Secretary


REPORT OF FRED. H. WINES, SECRETARY, TO THE ILLINOIS STATE BOARD OF PUBLIC CHARITIES, RESPECTING THE TENTH ANNUAL SESSION OF THE NATIONAL CONFERENCE OF CHARITIES, AT LOUISVILLE, KY., SEPT. 24–28, 1883.

GENTLEMEN:—I have the honor and pleasure of making the following report of my attendance upon the annual meeting of the Conference of Charities and Correction, which was in session at Louisville from the 24th to the 28th of September. During the greater part of this time the President of the Board, Dr. J. C. Corbus, and Mr. Whipp, my assistant in the office, were also present.

In several respects this has been the most interesting and profitable meeting which we have held, thus far. At Chicago, in 1879, there were but twelve States represented; at Cleveland, sixteen; at Boston, including the District of Columbia, there were nineteen; at Wisconsin, twenty-four; but at Louisville, thirty-three. We went there in hope of securing a full representation from the States south of the Ohio river, and in this we were scccessful. I think, too, that these States will send delegates regularly, hereafter. The number of delegates in attendance at Louisville was unusually large; it could not have been much, if any, less than two hundred persons from outside the bounds of Kentucky, and with the Kentuckians it probably reached three hundred. The *personnel* of the Conference was never finer than this year, nor the papers read better worth attention, nor the discussions freer from irrelevance and crudity of thought. Every moment of our time was occupied with the business of the session, which went forward rapidly and logically to its conclusion, and all who were there seemed to be satisfied with the result. From the people of Louisville and from the State officers of Kentucky we received every attention that we could have desired. I only regret that more of our own Board could not have been there.

MONDAY.

We met on Monday, in the evening, when addresses of welcome were made by Mayor Jacob, Gov. Knott and Senator Williams, which were responded to by Messrs. Sanborn, of Massachusetts, Vaux, of Pennsylvania, Mills, of North Carolina, and Knapp, of Florida.

My address, as President, was devoted to an exposition of our position and function as an organization apparently destined to exert considerable influence upon legislation and the future of charitable and correctional work in the United States.

TUESDAY.

Tuesday morning was devoted to hearing reports from the various States represented. There were so many of these, and the reports made were so unnecessarily long and minute, that the Conference was unable, during the four days that we were together, to hear from more than a part of them, but leave was given to any State not heard to file a written report for publication in the proceedings. The experience of this year led to the formation of a standing committee on Reports from States, whose duty it will be to see that reports are made in writing, and, as far as possible, placed in the hands of the committee at least one month in advance of our next meeting, so that the committee can prepare a summary of whatever is most interesting and important in them. This will not prevent the presentation of the reports separately, in addition.

We had two admirable reports on this day from the standing committees on the Work of the State Boards of Charity, by Bishop

Gillespie, and on Charity Organization in Cities, by Mr. McCulloch. Special prominence was given to the latter topic, which was discussed both in the afternoon and at night. Among those who participated in the debate were: Messrs. Putnam, of Boston, Fairchild, of New York, Walk and Garrett, of Philadelphia, Elliott and Barbour, of Detroit, Shattuck, of Cincinnati, and Mrs. Spencer, of Washington.

An interesting feature of the meeting on Tuesday was the address of Senator Vance, of North Carolina. The Conference listened with marked satisfaction, also to the addresses made by Rt. Rev. Father Bessonies, of Indiana, and Bishop Robertson, of Missouri.

WEDNESDAY.

Wednesday was given up to the consideration of the prison question. The standing committee on Crimes and Penalties reported, by its chairman, Mr. Z. R. Brockaway, Superintendent of the Reformatory at Elmira, New York, the only prison in the United States from which convicts are released on ticket-of-leave. Mr Brockaway's reputation as an able writer on this subject, as well as a most successful prison officer, is well known to you, but it seemed to me that in this report he surpassed anything which he has previously made public. The programme arranged by him for the day was very full, and included papers by Judge Young, of the Supreme Court of Minnesota, on the Reformatory Idea in Penal Treatment; by Miss Hall, of the Women's Prison, at Adrian, Michigan, on the Reformation of Criminal Girls; by Miss Mosher, formerly of the Women's Prison at Sherborne, Massachusetts, on Discipline in Prisons; by Gen. Brinkernoff, of Ohio, on what he called Post-Penitentiary Treatment of Criminals, that is to say, on police supervision of prisoners after their discharge; and by Judge Henry of the Supreme Court of Missouri, on Aid to Discharged Criminals. There was not as much time for discussion of these valuable essays as many desired. and some prison officers who were present, among whom I may mention Col. Lipscomb, of the South Carolina penitentiary, were not heard, which was the occasion of considerable regret.

Mrs. Barney, of Rhode Island, gave us a brief account of the efforts made by the Women's Christian Temperance Union to have female matrons appointed at the central police stations in cities.

Gov. Blackburn, of Kentucky, made an earnest and feeling speech, in which he pledged himself to devote himself for the remainder of his life to the cause of prison reform.

Other speakers were: Mr. Wilson, of Missouri, Gen. Taylor (a nephew of President Taylor), Chief of Police of the city of Louisville, Dr. Morris, of Baltimore, Dr. Cadwalader, of Philadelphia, Richard Vaux, Mrs. Cobb, of Milwaukee, Mrs. Beveridge, of Chicago, and some whose names I cannot at this moment recall.

But all will agree, I think, that the culminating point of interest was reached when, in the evening, Mr. George W. Cable, of New Orleans, the famous novelist, rose to read his carefully prepared attack upon the lessee prison system as administered at the South. For more than two hours he held his audience as if under a spell, while he quoted from the official reports of Southern prison officers and lessees, and drew from them inferences which, if they bear the test of examination, must, when they attract the notice of the Southern people, result in the abolition of the abuses which he depicted. When he took his seat, no one offered any defence of the system, but Gen. Anderson, of Kentucky, thrilled us by a fiery speech denouncing the wrongs perpetrated under it. Both Mr. Cable and Gen. Anderson were loudly applauded. It is said that Mr. Cable's article is to appear in the *North American Review.*

THURSDAY.

The Department of Justice sent a representative to the Louisville meeting—Mr. Haight, Inspector of Prisons in which United States prisoners are confined. There not having been time to hear him on Wednesday evening, he read, on Thursday morning, a brief but interesting account of the relation of the National Government to the prisoners of the country.

Dr. Bell, of Louisville, was introduced and spoke eloquently of the pleasure and advantage which our meeting had been to the people of the city and the State, and the influence which it would exert in the South.

Then followed a remarkable address by Rabbi Sonneschein, of St. Louis, on Hebrew Charity in the Middle Ages, delivered with great animation and enthusiastically applauded, especially the happy allusions made by him to Christianity, and his definition of charity as justice, the repayment of a gift.

The report of the standing committee on Preventive Work among Children was read by the Chairman, followed by a brief report by Mr. Coffin, of Indiana, on the International Congress at Paris composed of representatives of institutions and associations interested in child-saving. Judge Ferris, of Nashville, Tennessee, gave an account of his own extraordinary success in Placing Destitute and Homeless Children in Private Families, and Judge Lewis, also of Tennessee, read a paper entitled Industrial School Work. The session was closed by a paper from Mr. Letchworth, of New York, on the Classification and Industrial Employment of Destitute and Delinquent Children.

We spent the afternoon and evening at the House of Refuge, by invitation from the managers, who provided an elegant supper. We were invited, on the same evening, to the Central Lunatic Asylum, at Anchorage, and a part of the members went there, where they were equally hospitably entertained. The House of Refuge, under the superintendence of Mr. Caldwell, has reached a high point of excellence, and perhaps has no superior in this country, in many of the most important elements of successful reformatory work. The children were all assembled in the chapel, and were talked to, Mr. Mills, of North Carolina, bearing off the honors of the occasion by his quaint remarks on "the all-important subject of tar." The evening was spent in listening to accounts of reformatory institutions in New York, Pennsylvania and Ohio, in which Messrs. Fulton, Fay, Collins, Watson, Cooley, Douglas, and others, participated. There was not nearly time enough for all who had something to say, and the officers of reformatory institutions, of whom quite a number were present, organized themselves into a section on the following day, for the completion of the discussion.

FRIDAY.

Friday was a field-day, when all that had been overlooked or postponed found place. The first topic considered was the Care of the Chronic Insane, which was treated by Dr. Wardner, of the Illinois Southern Hospital for the Insane, at Anna. Among those who remarked on Dr. Wardner's paper were, Dr. Bryce of Alabama, and Dr. Griffin, of South Carolina.

Dr. Isaac L. Peet, the eminent principal of the New York Institution for the Deaf and Dumb, read a paper on the Education of Deaf Mutes. It happened that there were a good many teachers of the deaf present, from Ohio, Nebraska, Georgia, South Carolina, and elsewhere, and they, under the lead of Mr. Noyes, chairman of the standing committee on this subject, discussed Dr. Peet's paper. The statements of Mr. Gillespie with regard to the training of the ear by the use of the audiphone, awakened so much interest that the medical men present took it up, and Dr. Coomes, ot Louisville, described a novel instrument for testing the degree of sensibility in the auditory nerve by the aid of the microphone.

Dr. Walk, chairman of the standing committee on Preventive Medical Charities, read his report, which was accompanied by a paper on First Aid to the Injured, by Mr. Pine, of New York.

The last paper read was by Dr. Dewey, of Kankakee, on Building Plans for Public Institutions, concerning which it would have been appropriate to quote the Latin proverb, *Finis coronat opus.*

At the night session we listened to several admirable farewell speeches, three of which were by gentlemen of Louisville, namely: Mr. Caldwell, Congressman Willis, and the venerable Judge Bullock, who still retains his power to charm his hearers. The customary resolutions of thanks were adopted, some items of unfinished business were attended to, and the Conference adjourned.

RESOLUTIONS ADOPTED.

In this rapid résumé of our proceedings, which has, I trust, given some idea of the extent of ground covered and the variety and sustained interest of the papers and discussions, I have purposely omitted any reference to the action of the Conference upon other questions brought to its notice.

Concerning the death of Dr. H. B. Wilbur, of Syracuse, N. Y., the following resolutions, offered by Mr. Sanborn, were adopted by a rising vote :

"*Resolved*, That in the death of Dr. Hervey B. Wilbur, of New York, the Conference of Charities loses one of its earliest, most active and most valued members, who in his own specialty stood foremost, and whose wide acquaintance with other specialties, made his suggestions and criticisms of the highest worth to inspire, to correct, and to continue in action those practical means of administration, upon which the success of a public charitable system depends."

A committee was appointed to take suitable action respecting the International Penitentiary Congress, which has been called to meet next October, in the city of Rome, and brought in the following report, which was unanimously adopted:

"The Conference of Charities and Corrections, convened at Louisville, Ky., September 24-28, 1883, and representing the organized and individual charities and penal and reformatory institutions in the several States and in the District of Columbia, calls the attention of the President and Congress of the United States, and of the Governor and Legislature of each State, to the International Congress to convene at Rome, in Europe, October 15, 1884, and urges upon each the importance to this country, to our institutions, and to humanity, of a suitablere presentation of these States in that International Prison Congress.

"An American philanthropist, the late Dr. E. C. Wines, organized and put into action the International Prison Congress, and he took an active part in its meetings in London, in 1872, and again in Stockholm, in 1878, as Commissioner under appointment, and this country and Europe are reaping benefits resulting from the able and enlightened discussions at these meetings. Many important questions of criminal jurisprudence, of prison discipline, and of preventive measures will be before that Prison Congress for discussion and solution, and the United States, having institutions the most free, humane, enlightened and progressive of any in the world, cannot afford to withhold their aid or be absent from a congress dealing with such questions. Therefore this Conference of Charities and Corrections respectfully asks that Congress take due action to send to that coming meeting of the International Prison Congress three representatives, each from a different part of the country, to take part in its discussions and deliberations, and to bring to the United States the advanced wisdom and best results of its labors."

M. D. FOLLETT,

W. G. BULLOCK,

JNO. W. HENRY,

A. H. YOUNG,

Z. R. BROCKWAY.

Mr. Letchworth offered the following, which were also adopted:

"*Resolved, by the National Convention of Charities and Corrections of the United States*, That T. B. Ll. Baker, Esq., of Hardwicke Court, Gloucester, England, be invited to visit this country and attend the annual session of the Conference next year in the city of St. Louis.

"*Resolved*, That this action is not only an expression of our desire, but a recognition of the influence which Mr. Baker has had in molding, by his writings, and by his correspondence, the views of the Conference upon the important subjects which have come before us for our consideration."

ORGANIZATION.

St. Louis was chosen as the place of next meeting. Mr. Letchworth, of New York, was elected president for the ensuing year, and Bishop Robertson, of Missouri, vice president. Some changes were made in the list of corresponding secretaries, which was very much enlarged. Mr. Wright, of Wisconsin, retains his place as recording secretary; Mr. Milligan, of Pennsylvania, is promoted to be honorary secretary, in place of Mr. Sanborn; and the regular secretaries chosen are Dr. Hoyt, of New York, Mr. Caldwell, of Kentucky, and Mr. Hart, of Minnesota. The new standing committees and their chairmen are as follows:

On Reports from States: Fred. H. Wines, of Illinois.

On Charity Organization in Cities: C. S. Fairchild, of New York.

On the Organization and Management of Prisons and Penitentiaries: A. G. Byers, of Ohio.

On the Organization and Management of Reformatories and Houses of Refuge: A. E. Elmore, of Wisconsin.

On the Organization and Management of Poor-houses: H. H. Giles, of Wisconsin.

On Police System and Administration: F. L. Jenkins, of New York.

On Provision for the Chronic Insane: John B. Chapin, M.D., of New York.

On Provision for Idiots: Mrs. C. R. Lowell, of New York.

This is, of course, merely an outline of the proceedings, which were much more satisfactory than than they are here made to appear. I have not been able to name nearly all the speakers. It was noticeable that we had more speakers, and they spoke more briefly and to the point, than at any previous meeting, and there were but few who rose without having something of importance to say.

The excellent choir of the House of Refuge added much to our enjoyment.

Our special thanks are due to the local committee for their courtesy, forethought and aid.

With Mr. Letchworth as president, an equally good meeting, possibly a better, may be looked for at St. Louis. The time is not yet decided, but the sentiment of the members seemed to be in favor of May or June, rather than in the autumn.

All of which is respectfully submitted.

FRED. H. WINES, Secretary.

---

THE *St. Louis Medical and Surgical Journal* has, after late changes, the following editorial staff: Thos. Rumbold, M.D., editor and proprietor; John B. Keber, M.D., assistant editor; Frank M. Rumbold, business editor. Associate Editors:—H. Christopher, A.M., M.D., St. Joseph, Mo.; Wm. C. Byrd, M.D., Quincy, Ill.. A. E. Prince, M.D., Jacksonville, Ill.; LeGrand Atwood, M.D., St. Louis.

